# Low T-Cell Responses to Mitogen Stimulation Predicts Poor Survival in Recipients of Allogeneic Hematopoietic Stem Cell Transplantation

**DOI:** 10.3389/fimmu.2017.01506

**Published:** 2017-11-09

**Authors:** Michelle K. Yong, Paul U. Cameron, Monica A. Slavin, Allen C. Cheng, C. Orla Morrissey, Krystal Bergin, Andrew Spencer, David Ritchie, Sharon R. Lewin

**Affiliations:** ^1^Department of Infectious Diseases, Monash University and Alfred Hospital, Melbourne, VIC, Australia; ^2^The Peter Doherty Institute for Infection and Immunity, The University of Melbourne and Royal Melbourne Hospital, Melbourne, VIC, Australia; ^3^National Centre for Infections in Cancer, Peter MacCallum Cancer Centre, Melbourne, VIC, Australia; ^4^Victorian Infectious Diseases Service, Royal Melbourne Hospital at The Peter Doherty Institute for Infection and Immunity, Melbourne, VIC, Australia; ^5^Department of Haematology, Monash University and Alfred Hospital, Melbourne, VIC, Australia; ^6^Department of Clinical Haematology and Bone Marrow Transplant Service, The Royal Melbourne Hospital, Melbourne, VIC, Australia; ^7^Department of Medicine, University of Melbourne, Melbourne, VIC, Australia

**Keywords:** mitogen, biomarker, T-cell immunity, hematopoietic stem cell transplantation, mortality, prognosis, quantiferon-cytomegalovirus, transplantation

## Abstract

**Background:**

Successful engraftment and reconstitution of the innate and adaptive immune system are associated with improved outcomes in recipients of allogeneic hematopoietic stem cell transplantation (HSCT). A clinically meaningful and simple biomarker of immunosuppression could potentially assist clinicians in their decision-making. We aimed to determine the relationship between T-cell production of interferon gamma (IFN-γ) in response to phytohemagglutinin (PHA) to clinical outcomes in HSCT recipients.

**Methods:**

A prospective observational multicenter study of 73 adult allogeneic HSCT recipients was conducted in Melbourne, Australia. Eligible participants were >18 years and at risk of cytomegalovirus disease. T-cell responses to PHA were assessed at 3, 6, 9, and 12 months post-HSCT using the commercial quantiferon-cytomegalovirus assay, which quantifies IFN-γ production by ELISA following stimulation with PHA. A low response was defined as IFN-γ <0.5 IU/ml following stimulation with PHA.

**Results:**

At 3 months post-HSCT, high responses to PHA (median IFN-γ 7.68 IU/ml) were seen in 63% of participants and low responses to PHA (median IFN-γ 0.06 IU/ml) in 37%. IFN-γ responses to PHA were significantly associated with the severity of acute graft versus host disease (AGVHD) (spearman *r* = −0.53, *p* < 0.001) and correlated with blood lymphocyte count (spearman *r* = 0.52, *p* < 0.001). Twelve month overall survival was greater in individuals with high compared to low IFN-γ response to PHA at 3 months [92 vs. 62%, respectively, Cox proportional hazard ratio (HR): 4.12 95% CI: 1.2–13.7, *p* = 0.02]. Non-relapse mortality (NRM) was higher in individuals with low IFN-γ response to PHA (competing risk regression HR 11.6 *p* = 0.02). In individuals with no AGVHD compared to AGVHD and high IFN-γ response to PHA compared to AGVHD and low IFN-γ response to PHA, 12-month survival was 100 vs. 80 vs. 52%, respectively (log rank test *p* < 0.0001).

**Conclusion:**

Low IFN-γ response to PHA at the 3-month time-point following allogeneic HSCT was predictive of reduced 12-month overall survival, increased NRM, and reduced survival in recipients with AGVHD. Assessing IFN-γ response to PHA post-HSCT may be a clinically useful immune biomarker.

## Introduction

Despite recent advances in the field of transplantation, recipients of allogeneic hematopoietic stem cell transplantation (HSCT) remain profoundly immunocompromised and at high risk of early death from both infectious and non-infectious complications (1). Successful engraftment and immune reconstitution of the innate and adaptive immune systems have been associated with lower rates of infection, lower relapse rates, and improved survival (2). There is high clinical interest in finding appropriate immune biomarkers to predict clinical outcomes associated with immunosuppression in order to individualize clinical care (3–6). Following HSCT, clinicians often need to balance the risk of acute graft versus host disease (AGVHD) against infection, with both complications contributing significantly to transplant-related mortality. Potential candidate biomarkers used to predict mortality or AGVHD outcomes include levels of inflammatory cytokines (5), T-cell subset number (CD4+ or CD8+ T-cells) or rate of recovery (7, 8), C-reactive protein (9), interleukin receptors (3), ferritin (10), and functional immune assays (11).

We and other investigators have assessed the clinical utility of cytomegalovirus (CMV)-specific T-cell responses in recipients of HSCT in order to identify or predict CMV-related complications (12–14). The Quantiferon^®^-CMV is a simple *in vitro* interferon-gamma (IFN-γ) releasing functional assay, which measures whole blood T-cell activity against CMV antigens or phytohemagglutinin (PHA) without the need for peripheral blood mononuclear cell isolation (15). Here, we aimed to determine the clinical utility of measuring IFN-γ response to the positive mitogen control PHA, not CMV peptides. Previous studies in both solid and stem cell transplant recipients report that the Quantiferon assay is frequently “indeterminate” due to poor mitogen response as a result of immunosuppression (12, 16). Up to 38% of study samples were observed to have low T-cell IFN-γ cytokine function, particularly early posttransplantation when immunosuppression is greatest (12, 16). Because IFN-γ can be measured as a continuous variable, here, we evaluated T-cell responses to PHA in the Quantiferon-CMV assay as a continuous variable and assessed its relationship to clinical outcome in a prospective multicenter cohort study of adult allogeneic HSCT recipients.

## Materials and Methods

### Study Design

A prospective observational multicenter study of adult allogeneic HSCT recipients was conducted at two state-wide transplant centers in Melbourne, Australia; the Royal Melbourne Hospital and the Alfred Hospital between January 2011 and May 2014. Inclusion criteria were any participants receiving an allogeneic HSCT and at risk of CMV disease defined as concordant positive recipient (R)/donor (D) serology (R+/D+) or discordant recipient/donor serology (R+/D− and R−/D+) and have previously been reported (13). For this substudy of mitogen responses, only participants who had available study bloods taken at 3 months were eligible for inclusion. Exclusion criteria included low risk of CMV disease (R−/D−) and presence of CMV disease at the time of transplantation. The study period was 12 months from the date of transplantation and study specific bloods were taken 3, 6, 9, and 12 months post-HSCT. The study was approved by the human research ethics committees of The Alfred (HREC no. 339/10), Melbourne Health (MH 2010.290), and Monash University (CF11/0238-2011000078). Written informed consent was obtained from all participants.

### Transplant Protocol

Conditioning regimens for participants undergoing myeloablative HSCT included total body irradiation (day −4 to day −1) and cyclophosphamide of 60 mg/kg/day for 2 days (day −6 to day −5). Participants undergoing reduced intensity conditioning were treated with fludarabine 30 mg/m^2^, day −8 to −4 and melphalan 120–140 mg/m^2^, day −2. Conditioning regimen for T-cell depleted grafts included alemtuzumab or antithymocyte globulin (ATG) day −8 to day −4. Posttransplantation prophylaxis against graft versus host disease (GHVD) was with cyclosporine A plus either methotrexate or mycophenolate mofetil.

### Clinical Definitions

Clinical outcomes of interest included AGVHD, chronic graft versus host disease, relapse, non-relapse mortality (NRM), and 12-month overall survival. Diagnosis of AGVHD was defined using the histological biopsy results of the targeted organs involved and graded into categories I-IV according to published international guidelines (17). The primary cause of death was determined by the treating physician as recorded on the official death certificate.

### Immune Monitoring

Immune function was assessed using the commercially available Quantiferon^®^-CMV assay (QIAGEN, Germantown, MD, USA) (15). This *in vitro* whole blood assay, which quantifies production of IFN-γ following stimulation with (1) human leukocyte antigen (HLA) restricted CMV peptides, (2) PHA as a positive control, and (3) sterile phosphate buffered saline as a negative control. The assay was performed in accordance with manufacturer’s instruction as previously described (15, 18). In brief, 1 ml of whole blood is drawn into each of the three tubes and incubated overnight at 37°C. The supernatant was then harvested and ELISA performed for quantification of IFN-γ expressed as International units per milliliter. The final IFN-γ level was calculated by subtracting the IFN-γ expressed in the negative control tube from the PHA tube. The cut-off levels of IFN-γ that indicated a positive response to PHA was defined, as per manufacturer instructions. A low and high mitogen response to PHA was defined as IFN-γ < 0.5 IU/ml and IFN-γ ≥ 0.5 IU/ml, respectively. The maximum measured level of IFN-γ was 100 IU/ml and responses greater than this level were not accurately quantified.

### Statistical Analysis

Comparison of two categorical outcomes were calculated using chi square testing and Fisher’s exact test, as appropriate. A Wilcoxon rank sum test or Kruskal–Wallis test were used where groups of non-parametric continuous data were compared. We calculated the change in IFN-γ production in response to PHA over time in each participant using generalized estimating equations (GEE) modeling. Baseline was taken as the production of IFN-γ in response to PHA at 3 months posttransplant, and this model considered both variation at baseline as well as change over time (3–12 months) as an interaction with each group. SEs were calculated using the robust Huber–White sandwich estimator. Kaplan–Meier survival estimates and Cox proportional hazards model were calculated to assess all-cause mortality. Cumulative incidence curves with competing risks analysis were calculated for NRM where relapse was considered a competing event in accordance with statistical guidelines (19). A *p*-value <0.05 was considered to be statistically significant. Statistical analyses were performed using Stata 14.1 (Statacorp, College Station, TX, USA: StataCorp LP) and GraphPad Prism (v6; San Diego, CA, USA).

## Results

### Participant Demographics

**Figure 1:**
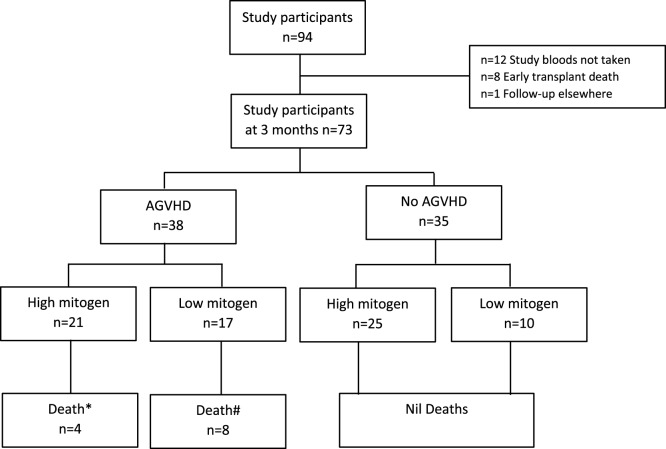
Study participant flow chart. *Death defined as 12-month all-cause mortality; attributable causes relapse (*n* = 3), infection (*n* = 1). ^#^Death defined as 12-month all-cause mortality; attributable cause graft versus host disease (*n* = 5), multi-organ failure (*n* = 2), hemorrhage (*n* = 1). AGVHD, acute graft versus host disease.

Ninety-four participants were recruited into the parent study and have been previously described (13). In brief, the most common indication for HSCT was acute myeloid leukemia (35%) and 63% of all transplants underwent myeloablative conditioning (13). Seventy-three participants of the parent study met this sub-study’s requirement for availability of a 3-month (post-HSCT) sample (see study flow chart Figure 1). Basic demographics for the 73 participants included into this sub-study are shown in Table 1.

**Table 1:** Participant characteristics.

	Participants	High IFN-γ response to phytohemagglutinin (PHA)	Low IFN-γ response to PHA	Uni-variate	Multi-variate

	*n* = 73 (%)	*n* = 46 (%)	*n* = 27 (%)	*p-*Value	*p-*Value
Age (years, median IQR)	50 (40–56)	48.5 (31–56)	53 (42–57)	0.2	–
Sex (male:female)	34:39	23:23	11:16	0.4	–
**Primary diagnosis**					
Acute myeloid leukemia	25 (34.2)	14 (30.4)	11 (40.7)		
Multiple myeloma	11 (15.1)	10 (21.7)	1 (3.7)		
Acute lymphoblastic leukemia	9 (12.3)	5 (10.9)	4 (14.8)		
Non-Hodgkins lymphoma	8 (11)	6 (13)	2 (7.4)		
Hodgkins disease	5 (6.9)	5 (10.9)	0	0.01	0.6
Myelodysplasia	5 (6.9)	2 (4.3)	3 (11.1)		
Chronic lymphocytic leukemia	4 (5.5)	4 (8.7)	0		
Chronic myeloid leukemia	3 (4.1)	0	3 (11.1)		
Myelofibrosis	2 (2.8)	0	2 (7.4)		
Blastic plasmacytoid dendritic	1 (1.4)	0	1 (3.7)		
**Type of conditioning**					
Myeloablative	47 (64.4)	26 (56.5)	21 (77.8)	0.07	0.09
Reduced-intensity conditioning	26 (35.6)	20 (43.5)	6 (22.2)		
**Graft source**					
Peripheral blood stem cell	62 (84.9)	42 (91.3)	20 (74.1)	0.1	–
Bone marrow	4 (5.5)	2 (4.3)	2 (7.4)		
Umbilical cord	7 (9.6)	2 (4.3)	5 (18.5)		
**Donor relationship**					
Sibling related	30 (41.1)	22 (47.8)	8 (29.6)	0.1	–
Volunteer unrelated donor	43 (58.9)	24 (52.2)	19 (70.4)		
**Conditioning**					
Fludarabine/melphalan	21 (28.8)	14 (30.4)	7 (25.9)		
Cyclophosphamide/TBI	13 (17.8)	8 (17.4)	5 (18.5)		
Busulphan/cyclophosphamide	10 (13.7)	6 (13)	4 (14.8)		
Fludarabine/TBI	10 (13.7)	10 (21.8)	0	0.05	0.4
Etoposide/TBI	5 (6.9)	4 (8.7)	1 (3.7)		
Fludarabine/cyclophosphamide	4 (5.5)	2 (4.35)	2 (7.4)		
Fludarabine/Cy/TBI/Thiotepa	4 (5.5)	1 (2.2)	3 (11.1)		
Fludarabine/cyclophosphamide/TBI	3 (4.1)	1 (2.2)	2 (7.4)		
Other	3 (4.1)	0	3 (11.1)		
**HLA match**					
Match	54 (74)	38 (82.6)	16 (59.3)	0.03	0.06
Minor mismatch	19 (26)	8 (17.4)	11 (40.7)		
**Graft versus host disease prophylaxis**					
Cyclosporin/methotrexate	46 (63)	29 (63)	17 (63)	0.5	–
Cyclosporin/mycophenolate	17 (23.3)	12 (26.1)	5 (18.5)		
Cyclosporin	8 (11)	4 (8.7)	4 (14.8)		
Other	2 (2.7)	1 (2.2)	1 (3.7)		
***In vivo* T cell depletion**					
Alemtuzumab	8 (11)	4 (8.7)	4 (14.8)	0.4	–
No Alemtuzumab	65 (89)	42 (91.3)	23 (85.2)		
***In vivo* T cell depletion**					
Antithymocyte globulin (ATG)	21 (28.8)	12 (26.1)	9 (33.3)	0.5	–
No ATG	52 (71.2)	34 (73.9)	18 (66.7)		
**Baseline cytomegalovirus category**					
Recipient+/donor+ (R+/D+)	33 (45.2)	23 (50)	10 (37)	0.3	–
Recipient+/donor	27 (37)	14 (30.4)	13 (48.1)		
Recipient	13 (17.8)	9 (19.6)	4 (14.8)		

*IFN-γ, interferon gamma; IQR, interquartile range; TBI, total body irradiation; Cy, cyclophosphamide; HLA, human leukocyte antigen*.

### Mitogen Responses Post-HSCT

Samples were available for evaluation at 207 study time-points; 3 months (*n* = 73), 6 months (*n* = 46), 9 months (*n* = 43), and 12 months (*n* = 45). Each participant contributed a median of 3 (range 1–4) study bloods. The median IFN-γ level following stimulation with PHA for study participants at 3, 6, 9, and 12 months were 2.05, 7.72, 30.5, and 16.63 IU/ml, respectively.

At 3 months post-HSCT, high mitogen responses were seen in 46 (63%) of participants and low mitogen responses were seen in 27 (37%) of participants (Table 1). The median [interquartile range (IQR)] mitogen IFN-γ level in high and low mitogen responders was 7.68 (2.43–33.83) IU/ml and 0.06 (0.01–0.17) IU/ml, respectively. In univariate analysis, the pretransplant factors associated with mitogen response at 3 months were the primary diagnosis (*p* = 0.01) and HLA matching (*p* = 0.03; Table 1). There was a trend toward significance with the conditioning regimen (*p* = 0.05) and intensity of conditioning (myeloablative versus. reduced intensity; *p* = 0.07). No associations were seen between mitogen responses at 3 months and age (*p* = 0.2), use of an unrelated donor graft (*p* = 0.1), nor T-cell depleting transplants using either alemtuzumab or ATG (*p* = 0.4 and *p* = 0.5, respectively). In a multivariate analysis, adjusted for the primary diagnosis, conditioning regimen, HLA matching, and intensity of conditioning, there was a trend toward HLA matching being an independent determinant of mitogen response at 3 months (*p* = 0.06; Table 1).

Interferon-gamma response to PHA significantly correlated to peripheral blood lymphocyte count at the time of study sampling (spearman *r* = 0.52, *p* < 0.001), but not to total white cell count or peripheral blood neutrophil count (Table 2). The median blood lymphocyte count in high and low mitogen responders at 3 months was 1 [0.7 − 1.7] × 10^9^/l and 0.6 [0.3 − 0.8] × 10^9^/l, respectively, *p* = 0.0001.

**Table 2:** Transplant-related outcomes.

	All participants	High IFN-γ response to mitogen	Low IFN-γ response to mitogen	*p-*Value

	*n* = 73 (%)	*n* = 46 (%)	*n* = 27 (%)	
**Blood parameters**				
Total white cell count (10^9^/l) median IQR	4.4 (3.2–6.4)	4.6 (3.6–6.3)	4.4 (3–6.7)	0.6
Neutrophil count (10^9^/l) median IQR	2.8 (1.8–4.3)	2.7 (1.9–3.7)	3 (1.7–5.6)	0.3
Lymphocyte count (10^9^/l) median, IQR	0.8 (0.5–1.3)	1 (0.7–1.7)	0.6 (0.3–0.8)	0.0001
**Acute GVHD**				
Nil	35 (48)	25 (54.3)	10 (37)	0.001
Grade I	16 (21.9)	12 (26.1)	4 (14.8)	
Grade II	12 (16.4)	9 (19.6)	3 (11.1)	
Grade III	7 (9.6)	0	7 (25.9)	
Grade IV	3 (4.1)	0	3 (11.1)	
**Acute GVHD treatment (*n* = 38)**				
Topical PNL only	9 (23.7)	8 (38.1)	1 (5.9)	0.04
PNL/cyclosporine or tacrolimus	28 (73.7)	13 (61.9)	15 (88.2)	
PNL/ATG/etanercept/pentostatin	1 (2.7)	0	1 (5.9)	
**Chronic GVHD**				
Nil	27 (38.6)	17 (37)	10 (41.7)	0.9
Limited	16 (22.9)	11 (23.9)	5 (20.8)	
Extensive	27 (38.6)	18 (39.1)	9 (37.5)	
**Death at 12 months**				
All-cause mortality	12 (16.4)	4 (8.7)	8 (29.6)	0.046
**Attributable death at 12 months**				
Graft versus host disease	5 (6.8)	0	5 (18.5)	
Relapse/progressive disease	3 (4.1)	3 (6.5)	0	
Multiorgan failure	2 (2.7)	0	2 (7.4)	
Hemorrhage	1 (1.4)	0	1 (3.7)	
Infection	1 (1.4)	1 (2.2)	0	

*PNL, prednisolone; ATG, antithymocyte globulin; GVHD, graft-versus-host-disease; IQR, interquartile range*.

**Figure 2:**
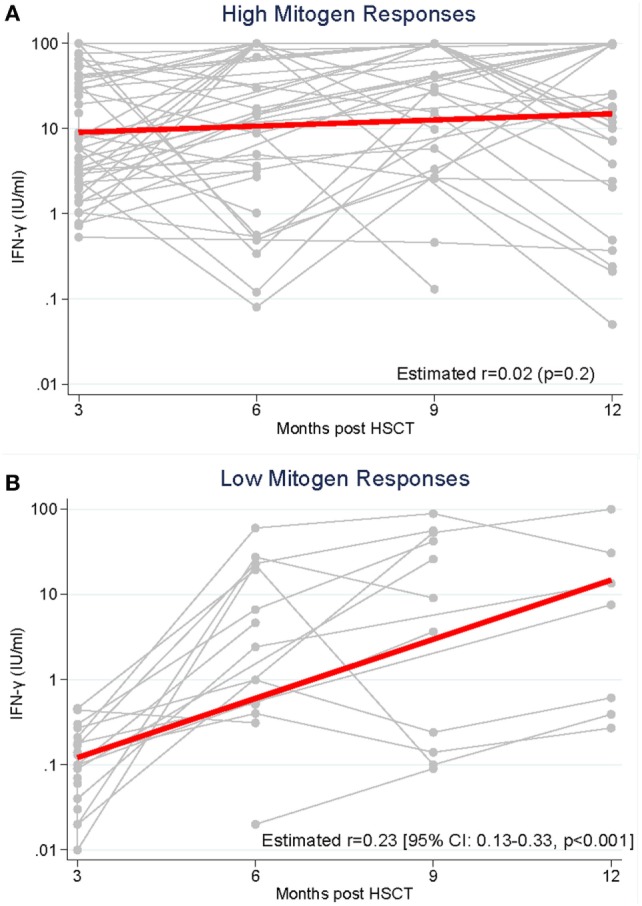
Production of interferon-gamma (IFN-γ) in response to phytohemagglutinin was measured from 3 to 12 months following hematopoietic stem cell transplantation (HSCT) in recipients with **(A)**. High mitogen responses (≥0.5 IFN-γ IU/ml) measured at 3 months post-HSCT and **(B)**. Low mitogen responses (<0.5 IFN-γ IU/ml) measured at 3 months.

We compared the longitudinal measurement of IFN-γ response to PHA between high and low mitogen responders (measured at 3 months post HSCT) over the study period (Figure 2). In high mitogen responders, the rate of IFN-γ response to PHA between 3 and 12 months post-HSCT did not change significantly (GEE estimated coefficient 0.02 *p* = 0.2; Figure 2A). In low mitogen responders, there was a significant increase in the IFN-γ response to PHA between 3 and 12 months (GEE estimated coefficient 0.23 *p* < 0.0001; Figure 2B).

### PHA Responses Predict Survival Outcomes

**Figure 3:**
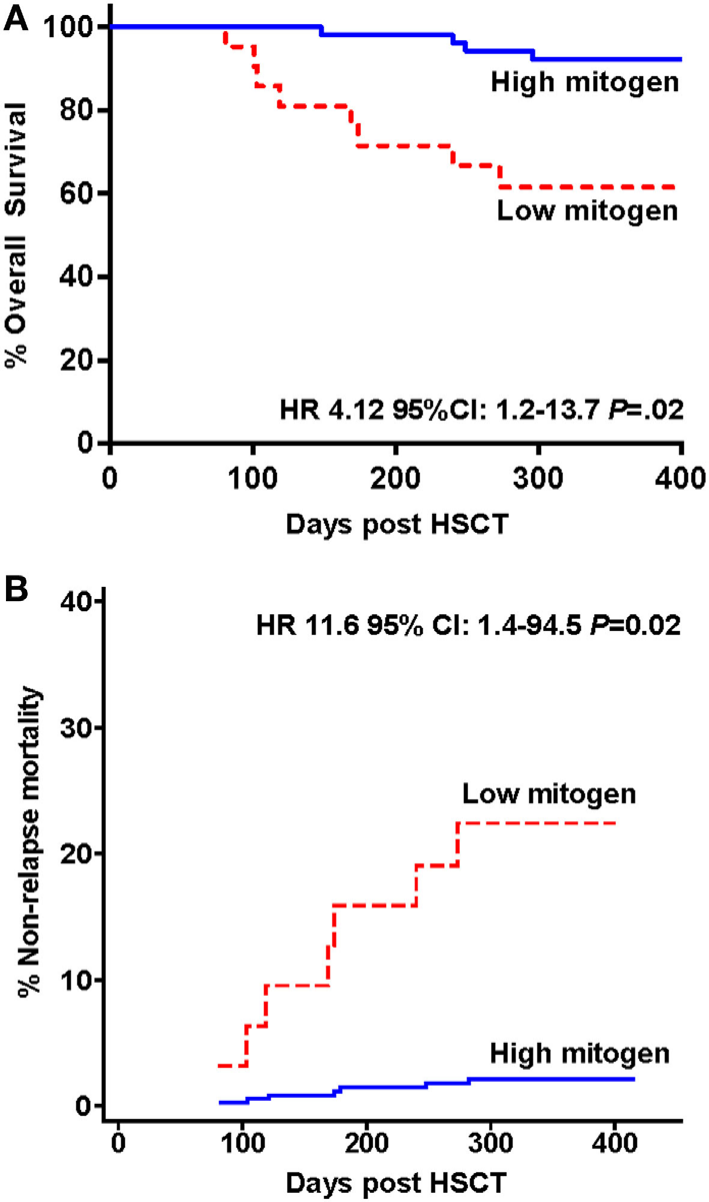
Survival and mortality in hematopoietic stem cell transplantation (HSCT) study participants stratified by low and high interferon-gamma production following phytohemagglutinin stimulation measured 3 months following HSCT. **(A)** Kaplan–Meier overall survival estimates and **(B)** cumulative incidence curve for non-relapse mortality are shown.

Twelve of the 73 study participants did not survive to 12 months (16.4%) with the attributable cause of death being AGVHD (*n* = 5), relapse of primary disease (*n* = 3), multi-organ failure (*n* = 2), hemorrhage (*n* = 1), and infection (*n* = 1), see Table 2. The overall 12-month survival significantly differed between individuals with a high versus low IFN-γ response to PHA at 3 months (92 vs. 62%, respectively, Mantel-Cox log-rank test *p* = 0.01; Figure 3A). The risk of death at any time in participants with a low vs. high response to PHA was significantly increased [Cox proportional hazards ratio (HR): 4.12 95% CI: 1.2–13.7, *p* = 0.02, Figure 3A]. After adjusting for the presence of AGVHD, the Cox proportional HR was 3.3 95% CI: 1–11, *p* = 0.052.

The cumulative incidence curve for NRM according to IFN-γ response to PHA is shown in Figure 3B. Participants with a low compared to high IFN-γ response to PHA at 3 months had a significantly increased risk of NRM at 12 months (competing risk analysis hazards ratio HR: 11.6 95% CI: 1.4–94.5, *p* = 0.02).

### PHA Responses Predict Survival in Individuals with AGVHD

**Figure 4:**
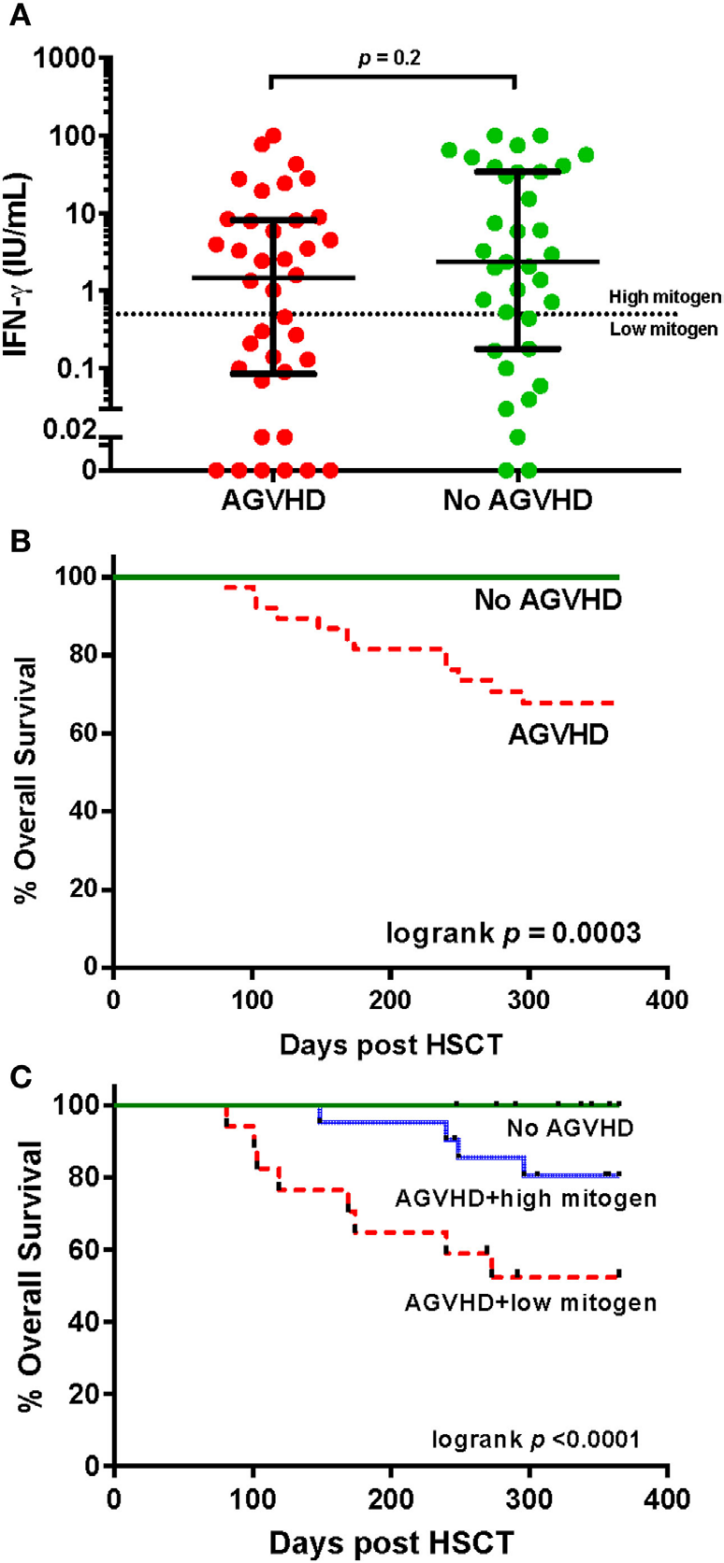
IFN-γ responses to phytohemagglutinin (PHA) and survival in individuals with and without acute graft versus host disease (AGVHD) following hematopoietic stem cell transplantation. **(A)** Comparison of the IFN-γ response (International units per milliliter) to PHA in participants with and without AGVHD at 3 months. The dotted horizontal line at an interferon-gamma (IFN-γ) level of 0.5 IU/ml represents the cut-off value determining high and low responses, according to the assay manufacturer. **(B)** Kaplan–Meier overall survival estimates for participants with and without AGVHD and **(C)** stratified by high and low IFN-γ response to PHA.

All study deaths (*n* = 12) occurred in HSCT recipients who developed AGVHD where AGVHD was either a direct (*n* = 5) or an indirect contributor to death (*n* = 7). AGVHD occurred in 38 of 73 (52%) participants at a median of 40 days (IQR 29–62) post HSCT. The development of AGVHD was not associated with IFN-γ response to PHA at 3 months (*p* = 0.15). There was no difference in the IFN-response to PHA at 3 months between those who did and did not develop AGVHD (median IFN-γ 1.47 vs. 2.34 IU/ml, respectively, *p* = 0.2 Figure 4A). However, the severity of AGHVD was strongly associated with IFN-γ response to PHA where all participants with grade III-IV AGVHD had low IFN-γ response to PHA (*p* = 0.001; Table 2). The treatment received for AGVHD was associated with IFN-γ response to PHA at 3 months. Participants receiving topical prednisolone for lower grade AGVHD (grade I-II) were more likely to have high IFN-γ response to PHA at 3 months (*p* = 0.04, Table 2). There was no significant difference in the time of onset of AGVHD between participants who had high and low IFN-γ response to PHA at 3 months (42 vs. 36 days, *p* = 0.2). Development of chronic GVHD was not associated with the IFN-γ response to PHA at 3 months post-HSCT (*p* = 0.9).

In participants who did not develop AGVHD, a low IFN-γ response to PHA at 3 months was observed in 28.6% of individuals (Figure 1). No patient deaths occurred in this group. There were no associations seen between IFN-γ response to PHA and T-cell depleting grafts using alemtuzumab or ATG in participants without AGVHD (Fisher’s exact *p* = 0.3).

Individuals with AGVHD compared to no AGVHD had reduced 12-month overall survival (68 vs 100%, respectively, Mantel log-rank test *p* < 0.0003; Figure 4B). The inclusion of IFN-γ response to PHA at 3 months post-HSCT in participants with AGVHD was able to predict and risk stratify participants with reduced 12-month overall survival. Survival rates in participants with no AGVHD compared to AGVHD with high IFN-γ response to PHA compared to AGVHD with low IFN-γ response to PHA was 100 vs. 80 vs. 52%, respectively (log-rank test *p* < 0.0001; Figure 4C). Of the 38 participants who developed AGVHD, the IFN-γ response to PHA at 3 months had 89% sensitivity and 69% specificity for NRM. The positive predictive value of developing NRM based on IFN-γ response to PHA at 3 months was 47% with a negative predictive value of 95%.

## Discussion

In this study, we prospectively evaluated IFN-γ production in response to PHA using a rapid and simple IFN-γ release assay and found that low IFN-γ response to PHA measured 3 months post-HSCT was predictive of lower 12-month survival and increased NRM. AGVHD severity was highly associated with IFN-γ response to PHA, and this relationship likely reflects the immunosuppressive effects of AGVHD and/or its treatment. Among HSCT recipients with AGVHD, the IFN-γ response to PHA also predicted 12-month survival. A low IFN-γ response to PHA could allow individualized tailoring of immunosuppression to potentially prevent poor transplant-related outcomes.

A biomarker that could accurately assess an individual’s level of immunosuppression is of high clinical interest (20, 21). Potential biomarkers should ideally be easy to perform, be reproducible, and accurately predict outcomes such as AGVHD (3, 5), infection (9, 22), and survival (3, 9). Candidate biomarker targets have included T-cell subsets (7, 23), functional immune assays (11, 23), inflammatory cytokines (22), and interleukin receptors (5). T-cell subset numbers, particularly higher CD8+ T-cells but not CD4+ T-cells, at day 90 were recently observed to predict enhanced survival in a cohort of HSCT recipients but immune function was not assessed (7). The Cylex ImmuKnow^®^ is another immune monitoring assay, which after stimulation with PHA measures intracellular adenosine triphosphate levels in CD4+ T-cells (24). Several studies in the HSCT population did not demonstrate any additional clinical value of the ImmunKnow assay (11, 25, 26). More complex biomarkers such as T-cell immunoglobulin and mucin-domain containing 3 (TIM3), interleukin-6, soluble tumor necrosis factor receptor 1, and plasma suppression of tumorigenicity 2 have shown some promise in predicting AGVHD and NRM (3, 5).

Both the Quantiferon-CMV and Quantiferon-TB (tuberculosis) assay include a PHA control and, therefore, both assays could potentially be used to risk stratify individuals at high risk of poor survival. Advantages of the Quantiferon assay are that it is simple to perform in a routine diagnostic laboratory, is standardized, and results could be available in real time to help guide clinical decisions. These assay characteristics address some of the concerns that candidate biomarker assays are too complicated or technically sophisticated to be used in clinical practice (20). Here, we showed the assay had some predictive value which could potentially impact on clinical decision-making. A major limitation in assessing individual biomarkers has been the clinical interpretation, since statistically significant differences do not necessarily translate into changes in clinical management or diagnostic accuracy (20).

There are several limitations to this study. The overall size of the cohort was relatively small and the study results need further validation in a larger cohort. We assessed clinical outcomes at 12 months post-HSCT, but a longer duration of follow-up should be examined to determine durable clinical benefits. As we only assessed a single cytokine IFN-γ using whole blood, the exact cellular source of IFN-γ was not evaluated and could have been from either T-cells or natural killer cells. Furthermore, the assay evaluated in this study did not assess the quantity or phenotype of the T-cell response.

In conclusion, low IFN-γ responses to PHA stimulation measured 3 months following allogeneic HSCT was predictive of increased NRM and reduced 12-month overall survival, particularly in recipients who developed AGVHD. This IFN-γ release assay is a simple and easy to perform assay with the capacity for high throughput. Further validation of the clinical utility of this assay in a larger HSCT cohort is warranted.

## Ethics Statement

This study was carried out in accordance with the recommendations of the human research ethics committees of The Alfred (HREC no 339/10), Melbourne Health (MH 2010.290), and Monash University (CF11/0238-2011000078) with written informed consent from all subjects. All subjects gave written informed consent in accordance with the Declaration of Helsinki. The protocol was approved by The Alfred, Melbourne Health, and Monash University human research ethics committees.

## Author Contributions

MY, PC, and SL designed the study. MY, MS, CM, DR, AS, and KB acquired the data. MY performed the laboratory studies and wrote the manuscript. MY, PC, SL, and AC assisted with data interpretation and AC provided statistical support. All authors contributed intellectual knowledge and revised the final manuscript. SL provided over-arching supervision and support.

## Conflict of Interest Statement

The authors declare that the research was conducted in the absence of any commercial or financial relationships that could be construed as a potential conflict of interest. The reviewer BE-V and handling editor declared their shared affiliation.
